# Molybdenum Carbide Nanoparticles Coated into the Graphene Wrapping N‐Doped Porous Carbon Microspheres for Highly Efficient Electrocatalytic Hydrogen Evolution Both in Acidic and Alkaline Media

**DOI:** 10.1002/advs.201700733

**Published:** 2018-01-03

**Authors:** Huifang Wei, Qiaoya Xi, Xi'an Chen, Daying Guo, Feng Ding, Zhi Yang, Shun Wang, Juan Li, Shaoming Huang

**Affiliations:** ^1^ Key Laboratory of Carbon Materials of Zhejiang Province College of Chemistry and Materials Engineering Wenzhou University Wenzhou 325035 P. R. China; ^2^ School of Materials and Energy Guangdong University of Technology Guangzhou Guangdong 510006 P. R. China

**Keywords:** electrocatalytic hydrogen evolution, graphene wrapping N‐doped porous carbon microspheres, molybdenum carbide, spray‐drying

## Abstract

Molybdenum carbide (Mo_2_C) is recognized as an alternative electrocatalyst to noble metal for the hydrogen evolution reaction (HER). Herein, a facile, low cost, and scalable method is provided for the fabrication of Mo_2_C‐based eletrocatalyst (Mo_2_C/G‐NCS) by a spray‐drying, and followed by annealing. As‐prepared Mo_2_C/G‐NCS electrocatalyst displays that ultrafine Mo_2_C nanopartilces are uniformly embedded into graphene wrapping N‐doped porous carbon microspheres derived from chitosan. Such designed structure offer several favorable features for hydrogen evolution application: 1) the ultrasmall size of Mo_2_C affords a large exposed active sites; 2) graphene‐wrapping ensures great electrical conductivity; 3) porous structure increases the electrolyte–electrode contact points and lowers the charge transfer resistance; 4) N‐dopant interacts with H^+^ better than C atoms and favorably modifies the electronic structures of adjacent Mo and C atoms. As a result, the Mo_2_C/G‐NCS demonstrates superior HER activity with a very low overpotential of 70 or 66 mV to achieve current density of 10 mA cm^−2^, small Tafel slope of 39 or 37 mV dec^−1^, respectively, in acidic and alkaline media, and high stability, indicating that it is a great potential candidate as HER electrocatalyst.

As a clean and sustainable energy source, hydrogen has been considered as one of the most alternatives to fossil fuels.^1^ The evolution of hydrogen through electrocatalytic splitting water is one of the important strategies for hydrogen production.^2^ Although the Pt‐based materials have been proven to be the most efficient electrocatalysts for hydrogen evolution reaction (HER), the high cost, limited supply, and poor durability hinder their global‐scale application.^3^, ^4^ Therefore, much effort has been dedicated to develop robust nonnoble‐metal HER catalysts,^5^, ^6^, ^7^, ^8^, ^9^, ^10^, ^11^, ^12^, ^13^, ^14^, ^15^, ^16^, ^17^, ^18^, ^19^, ^20^, ^21^, ^22^ such as cobalt‐, nickel‐, iron‐, tungsten‐, and molybdenum‐based materials.

In recent years, Mo_2_C has been exploited as one of the promising HER electrocatalysts due to its Pt‐like features and low‐cost. However, Mo_2_C obtained at high temperature usually suffers the inevitable aggregation,^23^ which leads to the less exposure active sites, thus compromising the HER performance. To tackle the drawback, various conductive carbon materials, such as graphene, carbon nanotube, porous carbon, etc., were introduced as framework for supporting Mo_2_C to reduce its aggregation and improve conductivity.^24^, ^25^, ^26^, ^27^, ^28^, ^29^, ^30^ Recently, the electrochemically active N‐doped nanocarbon matrix for Mo_2_C has been proved to be effective for improving its electrocatalytic activity, mainly due to that the N‐dopant could interact with H^+^ better than C atoms for enhanced H^+^ adsorption and favorably modify the electronic structures of adjacent Mo and C atoms for improved H desorption from Mo—H.^31^, ^32^, ^33^, ^34^, ^35^, ^36^, ^37^, ^38^, ^39^, ^40^, ^41^, ^42^, ^43^, ^44^ Despite these progresses, exploring the suitable precursors to uniformly hybridize Mo and carbon source to perform the controllable preparation of well‐defined Mo_2_C nanostructure with an N‐doped nanocarbon framework is still great demand.

Herein, we provide a simple protocol for synthesis of ultrafine Mo_2_C nanoparticles uniformly anchored into graphene wrapping N‐doped porous carbon microspheres (Mo_2_C/G‐NCS) using low cost chitosan and ammonium molybdate tetrahydrate (AM) as carbon and Mo source, respectively (**Scheme** **1**, see in the Experimental Section for detail). By virtue of the electrostatic interaction of Mo_7_O_24_^−^ and the —NH_3_^+^ group derived from the long chain chitosan molecular in acetic acid medium (Figure S1, Supporting Information), the uniform hybrid Mo and carbon source can be achieved, thus ensuring the well‐regulated Mo_2_C‐embedded into N‐doped carbon nanostructure. The particle average size increases when the AM is added into chitosan solution, which indirectly verifies the existence of electrostatic interaction (Figure S2, Supporting Information). Due to the such designed structure with N‐doped carbon microsphere as carbon matrix and graphene wrapping for fast electron transfer, ultrafine Mo_2_C nanoparticles for sufficient catalytic sites, and porous structure for easy mass transfer, the resulting material acts as highly active and stable nonplatinum HER electrocatalyst with a very low overpotential of 70 or 66 mV to achieve current density of 10 mA cm^−2^, small Tafel slope of 39 or 37 mV dec^−1^, respectively, in acidic and alkaline media, which is one of most active Mo‐based HER electrocatalysts and comparable to the commercial 20 wt% Pt/C catalyst.

**Scheme 1:**
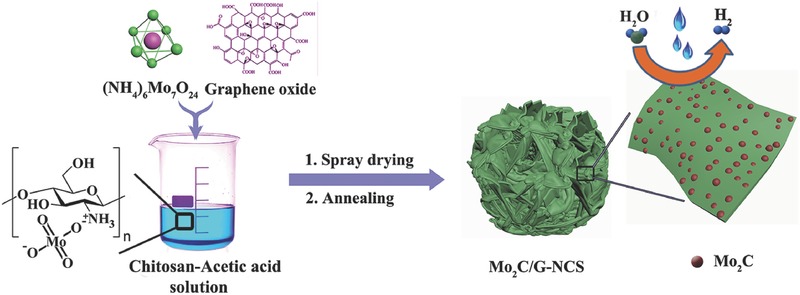
Schematic illustration of the procedure for preparing Mo_2_C/G‐NCS.

The morphology and microstructure of as‐prepared Mo_2_C‐based composites were first observed by field emission scanning electron microscopy (FESEM) and transmission electron microscopy (TEM). As can been seen from **Figure** **1**a, the product Mo_2_C/G3‐NCS750 is composed of uniform microspheres with rough surface and porous texture. On the contrast, the corresponding products Mo_2_C/NCS750 without the addition of graphene oxide and Mo_2_C/NC750 without the spray‐drying show relatively smooth and compact surface (Figure S3, Supporting Information). This indicates that the spray drying and addition of graphene oxide could cause into a loose, porous texture, thus not only increasing the electrolyte–electrode contact points and exposing the more active sites but also providing efficient electron transport and mass transfer. This is also verified by nitrogen adsorption/desorption isotherms (Figure S4, Supporting Information). The sample Mo_2_C/NC750 has little adsorption of nitrogen gas, indicating its extremely small specific surface area and the absence of pores. By contrast, the adsorption amount of nitrogen gas obviously increases for Mo_2_C/G3‐NCS750, indicating that it possesses a larger Brunner−Emmet−Teller (BET) surface area than that of Mo_2_C/NC750. From the TEM image, the graphene wrapping the microspheres to form the rugged surface is also observed clearly. The high‐resolution TEM (HRTEM) image shows that ultrafine nanoparticles with a size ≈4 nm are embedded into carbon matrix (Figure 1c; Figure S5, Supporting Information), which is consistent with the observation from the bright and dark field TEM image (Figure S6, Supporting Information). The apparent lattice fringes with a distance of 0.23 and 0.34 nm are assigned to crystallographic planes (101) of hexagonal Mo_2_C and (002) of graphene, respectively. X‐ray energy‐dispersive spectroscopy (EDS) elemental mapping (Figure 1d–g) indicates the C, Mo, N, and O atoms are uniformly distributed into the hybrids, which verifies that there is a homogeneous coating of Mo_2_C nanoparticles. N atoms are originated from the amino group of chitosan and NH_4_^+^ of (NH_4_)_6_Mo_7_O_24_.

**Figure 1:**
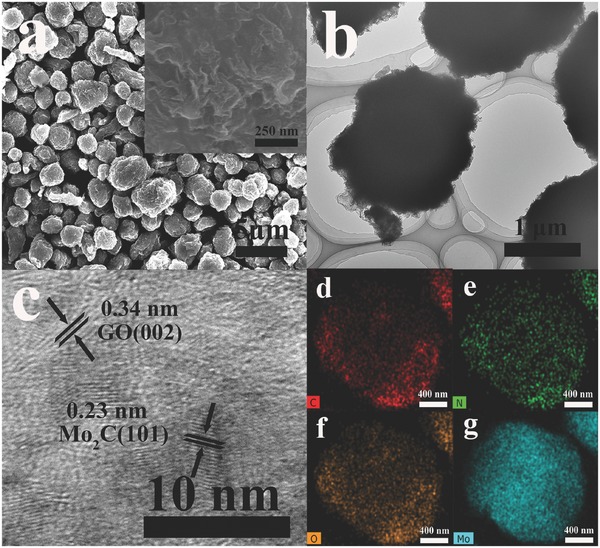
a) SEM images at different magnifications, b,c) TEM images at different magnifications and d–g) the corresponding EDS elemental mapping of Mo_2_C/G3‐NCS750.

The crystalline phase composition of as‐prepared Mo_2_C‐based materials yielded at different condition was determined by powder X‐ray diffraction (**Figure** **2** ). When the precursor is pyrolyzed, the corresponding product Mo_2_C/G3‐NCS750 clearly shows the characteristic diffraction peaks at 34.5°, 38.0°, 39.6°, 52.3°, 61.9°, 69.8°, 72.8°, 75.0°, and 76.0°, attributing to the (100), (002), (101), (102), (110), (103), (200), (112), and (201) planes of a hexagonal β‐Mo_2_C (JCPDS 65‐8766), respectively. No addition peak is observed except for a weak diffraction peaks at 42.6° corresponding to (100) plane of graphite. The particle size was evaluated by Scherrer equation, and the average size of Mo_2_C particles is around 4.5 nm (see the calculation details in the Supporting Information). These results agree well the HRTEM observation. A similar X‐ray diffraction (XRD) pattern is observed for the Mo_2_C/G3‐NCS850, suggesting the similar crystalline structure. The average size of Mo_2_C particles increases to 6.5 nm calculated according the Scherrer equation (see the calculation details in the Supporting Information) when the pyrolysis temperature is elevated to 850 °C, which is consistent with the size of ≈6 nm judged by the observation on the HRTEM image (Figure S7, Supporting Information). When the annealing temperature decreases to the 650 °C, no characteristic diffraction peak of the Mo_2_C is observed for Mo_2_C/G3‐NCS650. These results offer strong evidence of a solid‐state reaction between (NH_4_)_6_Mo_7_O_24_ and chitosan at about 750 °C. Other than the characteristic diffraction peaks of the Mo_2_C, the (002) diffraction peak of MoO_2_ is observed for the product Mo_2_C/G5‐NCS750, where the amount of graphene oxide increases (Figure S8, Supporting Information). The materials Mo_2_C/NC750 and Mo_2_C/NCS750 also show the similar crystalline structure (Figure S8, Supporting Information).

**Figure 2:**
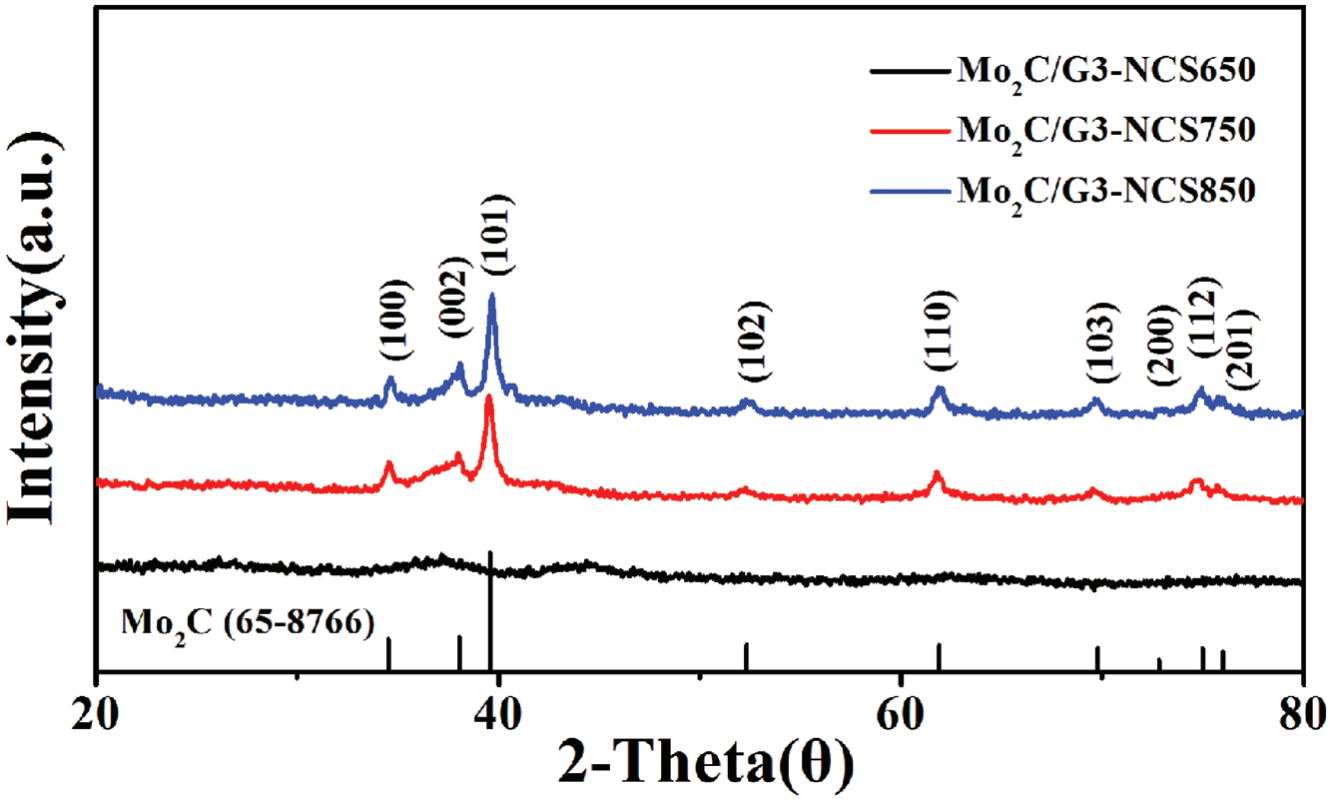
XRD patterns of all the as‐prepared composites.

Since the HER performance of transition‐metal‐based materials is closely corrected with the chemical environment of the metal, the surface electronic state and composition of Mo_2_C/G3‐NCS750 were further characterized by X‐ray photoelectron spectroscopy (XPS). The survey spectrum (**Figure** **3**a) displays the obvious signals of the atom Mo and C, as well as the atom N, indicating that N has been successfully introduced into Mo_2_C/G3‐NCS750 as expected. The high resolution Mo 3d spectrum (Figure 3b) can be well‐fitted into four pairs of peaks, indicating that four oxidation states for Mo species (+2, +3, +4, and +6) exist on the surface of Mo_2_C nanoparticles. The peaks at binding energies of 230.4/234.2 and 232.2/235.9 eV are ascribed to Mo^4+^ and Mo^6+^ species, which is attributed that the surface of Mo_2_C NPs can be readily oxidized to molybdenum oxides when exposed to air.^24^, ^25^, ^30^ Mo^2+^ with the binding energies of 228.3/231.4 eV is assigned as carbides, which is known to be served as active sites for HER.^23^, ^24^, ^25^ From the thermal gravimetric analysis of Mo_2_C/G3‐NCS750 composite, the content of Mo_2_C is about 69.7% (Figure S9, Supporting Information). The peaks at binding energies of 229.1/232.8 eV corresponding to Mo^3+^ for nitrides suggest N occupying part of C sites in Mo_2_C nanoparticles.^37^, ^39^, ^41^, ^42^ The deconvolution analysis of the detailed N1s spectrum (Figure 3c) displays the presence of pyridinic N (398.4 eV), graphitic N (401.7 eV), and N—Mo (396.4 eV).^37^, ^42^ Among these type of N‐doping, the pyridinic N is the main nitrogen species in Mo_2_C/G3‐NCS750, which is beneficial to the HER.^32^, ^34^, ^37^, ^39^ The presence of low level (less than 4%) of N—Mo species further confirms that N is doped into molybdenum carbide, which is in good agreement with the analysis of Mo 3d spectrum. This part of N‐doping as an electron‐rich dopant could downshift the density of empty d‐band in Mo_2_C, and thus weaken Mo—H strength.^37^, ^39^, ^41^, ^42^ No diffraction signals of nitride are identified by XRD analyzer, indicating that few N occupy part of C sites in Mo_2_C, which agrees well the above analysis of N1s XPS spectrum. The deconvolution analysis of the high‐resolution C1s spectrum is also consistent with the expected structure of Mo_2_C/G3‐NCS750 (Figure 3d).

**Figure 3:**
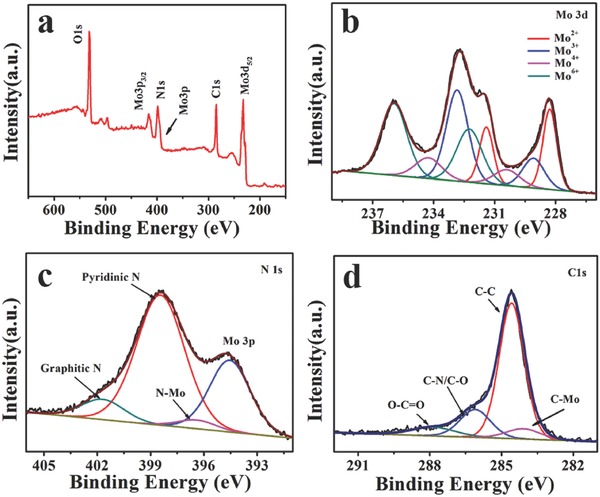
a) The wide, b) Mo 3d, c) N1s, and d) C1s XPS spectra of Mo_2_C/G3‐NCS750.

The electrocatalytic HER performance of the as‐prepared Mo_2_C‐based materials was first investigated using three‐electrode electrochemical configuration in 0.5 m H_2_SO_4_ solution, as well as the commercial catalyst 20% Pt/C. **Figure** **4** displays the corresponding linear sweep voltammetry (LSV) curves. As expected, the Pt/C catalyst is highly active toward HER in acidic electrolyte with a near‐zero onset overpotential. It is exciting that Mo_2_C/G3‐NCS750 exhibits a very small overpotential of 70 mV to drive a current density of 10 mA cm^−2^. Furthermore, the current density can reach to 75 mA cm^−2^ at an overpotential of 150 mV, which is even larger than the value obtained at η = 200 mV for many Mo_2_C‐based electrocatalysts reported. These results represent that as‐prepared Mo_2_C/G3‐NCS750 is one of the currently most efficient Mo_2_C‐based HER electrocatalysts in acidic electrolyte (Table S1, Supporting Information). On the contrary, Mo_2_C/NC750 and Mo_2_C/NCS750 need the overpotential of 97 and 82 mV, respectively, to achieve a current density of 10 mA cm^−2^. The enhancement on the HER performance is believed to be attributed that graphene‐wrapping improves electrical conductivity, and porous structure increases the electrolyte–electrode contact points and lower the charge transfer resistance, thus exposes more active sites, which is evidenced by the electrochemically active surface area (ECSA). To evaluate the ECSA of these as‐prepared electrocatalysts, the cyclic voltammetry (CV) was measured in the region from 0.2 to 0.4 V at scan rate varying from 20–180 mV s^−1^. The calculated electrochemical double‐layer capacitance (*C*_dl_) of Mo_2_C/G3‐NCS750 (46.3 mF cm^−2^) is ≈3 times larger than Mo_2_C/NC750 (11.9 mF cm^−2^) and Mo_2_C/NCS750 (13.3 mF cm^−2^) (Figure S10, Supporting Information). If we suppose a standard value of 40 µF cm^−2^,^37^ the ECSA of Mo_2_C/G3‐NCS750 is estimated to ≈91.3 cm^3^ g^−1^ (see the calculation details in the Supporting Information). ESCA of Pt/C electrocatalyst is also calculated (≈71.0 cm^3^ g^−1^), and the polarization curves of Mo_2_C/G3‐NCS750 and Pt/C are normalized by ESCA (Figures S11 and S12, Supporting Information). From the Figure S12 (Supporting Information), the Mo_2_C/G3‐NCS750 still exhibits excellent HER performance, which is comparable to the Pt/C electrocatalyst.

**Figure 4:**
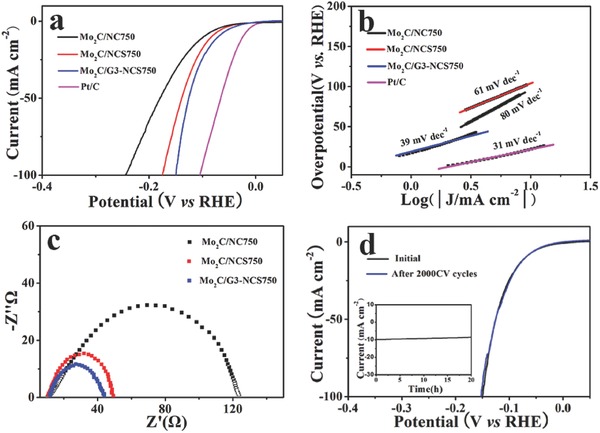
a) LSV curves and b) the corresponding Tafel plots of Mo_2_C/NC750, Mo_2_C/NCS750, Mo_2_C/G3‐NCS750, and Pt/C. c) EIS Nyquist plots collected at a bias voltage of −80 mV for Mo_2_C/NC750, Mo_2_C/NCS750, and Mo_2_C/G3‐NCS750. LSV curves initial and after 2000 CV of d) Mo_2_C/G3‐NCS750 (chronoamperometry test as the inset) in 0.5 m H_2_SO_4_ solution.

In control experiments, we first investigate the influence on the performance of the mass ratio between AM and chitosan (Figure S13, Supporting Information). When a mass ratio of AM and chitosan is 1:1, the corresponding product Mo_2_C/NC750 displays best HER activity. The effect of the carbonization temperature and amount of graphene oxide on the electrocatalytic properties were further evaluated (Figure S14, Supporting Information). Further increasing the annealing temperature to 850 °C, the corresponding electrocatalyst Mo_2_C/G3‐NCS850 shows a slightly larger onset overpotential. The decline in performance is believed to be ascribed to the increase of Mo_2_C particle size, which is verified by the above analysis of XRD and HRTEM. A significant increase in overpotential is also observed for Mo_2_C/G3‐NCS650, indicating its poor HER performance, which is mainly attributed that Mo_2_C could not be formed at an annealing temperature of 650 °C according to the XRD analysis. This result implies that the HER activity is mainly originated from the Mo_2_C species. Mo_2_C/G5‐NCS750 containing more amount of graphene does not exhibit better performance. A probable reason is that more graphene is coated on molybdenum carbide to limit exposure to its active sites. These results suggest that the synthetic conditions for fabrication of Mo_2_C/G3‐NCS750 with high HER performance have been optimized.

As we known, the Tafel slope indicates the intrinsic activity of HER electrocatalysts, and smaller Tafel slope means faster HER rate with the increased overpotentials. In the circumstances, Tafel slope originated from the polarization curves is fitted to the Tafel equation (η = *b* log (*j*) + *a*, where *j* is the current density and *b* is the Tafel slope), and shown in **Figure** **5**b. The Pt/C displays a Tafel slope of 31 mV dec^−1^, which is consistent with the reported values,^24^ thus supporting the reliability of our electrochemical measurements. It is notable that Mo_2_C/G3‐NCS750 achieves a small tafel slope of 39 mV dec^−1^, which is much lower than those of Mo_2_C/NC750 (80 mV dec^−1^) and Mo_2_C/NCS750 (61 mV dec^−1^), and most of the reported Mo_2_C‐based electrocatalysts (Table S1, Supporting Information). The smaller Tafel slope indicates the faster proton discharge kinetic and superior HER activity for Mo_2_C/G3‐NCS750 sample. According to the classical two‐electron‐reaction pattern, the HER in the acidic media generally proceeds in followed two steps. First, the Volmer reaction (H_3_O^+^ + e^−^ → H*ads + H_2_O) is assigning to the H^+^ adsorption step at the Tafel slope of 120 mV dec^−1^. Second, the Heyrovsky reaction (H*ads + H_3_O^+^ + e^−^ → H_2_ + H_2_O) is the desorption step, or the Tafel reaction (H*ads + H*ads → H_2_) is a recombination step at the Tafel slope of 40 mV dec^−1^ or 30 mV dec^−1^, respectively. In the case, the tafel slope of 39 mV dec^−1^ demonstrates that Mo_2_C/G3‐NCS750 catalyzed HER proceeds by a Volmer–Heyrovsky mechanism, where the electrochemical desorption of hydrogen is the rate‐limiting step. The *j*_0_ value of Mo_2_C/G3‐NCS750 was determined to be 0.33 mA cm^−2^, outperforming many Mo_2_C‐based HER electrocatalysts reported in the literature, such as MoDCA‐5 (0.179 mA cm^−2^),^39^ P‐Mo_2_C@C NWs (0.18 mA cm^−2^),^24^ etc. Electron impedance spectroscopy (EIS) was conducted to further probe the catalytic behavior of these as‐prepared electrocatalysts, and the corresponding Nyquist plots are shown in Figure 4c. Since the semicircle in the low‐frequency region typically means the charge transfer resistance, the smaller *R*_ct_ value implies that a faster electrode kinetic is occurred for the Mo_2_C/G3‐NCS750 catalyst. Mo_2_C/NC750 and Mo_2_C/NCS750 display the higher impedance, which is well in agreement with the analysis result of the Tafel slopes. In addition, the only one semicircle also indicates that HER proceeds by a Volmer–Heyrovsky mechanism for these presented electrocatalysts.

**Figure 5:**
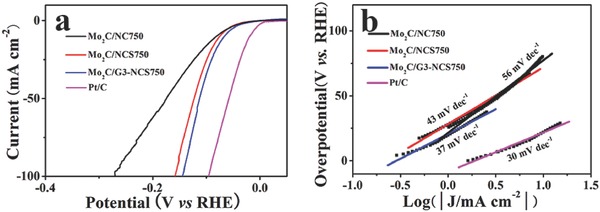
a) LSV curves and b) the corresponding Tafel plots of Mo_2_C/NC750, Mo_2_C/NCS750, Mo_2_C/G3‐NCS750, and Pt/C in 1 m KOH solution.

Considering that the long‐term stability is another key factor of the catalytic performance of a HER electrocatalyst, the durability of Mo_2_C/G3‐NCS750 catalyst was evaluated by 2000 cycles of CV scanning from 0.05 to −0.4 V at a scan rate of 100 mV s^−1^ in acidic electrolyte. As can be seen from Figure 4d, the almost same polarization curves of Mo_2_C/G3‐NCS750 before and after 2000 CV cycles are observed, suggesting its excellent stability. Meanwhile, the chronoamperometry test of Mo_2_C/G3‐NCS750 for 20 h at η = 70 mV in acidic electrolyte was also performed. Only ≈10% catalytic current decay of Mo_2_C/G3‐NCS750 demonstrates its high stability under corrosive acidic medium. As mentioned, the crystalline phase composition of Mo_2_C/G3‐NCS750 after continuously producing hydrogen for 20 h remains unchanged, which is evidenced by the XRD analysis (Figure S15, Supporting Information).

Since water splitting is often occurred in alkaline solution for practical applications, the HER performance of as‐prepared molybdenum‐based catalyst is further evaluated in 1 m KOH. The HER activity of as‐prepared the Mo_2_C‐based catalysts (Mo_2_C/G3‐NCS750, Mo_2_C/NCS750, and Mo_2_C/NC750) is slightly enhanced in alkaline solution instead of acidic medium, and their overpotential to achieve 10 mA cm^−2^ is reduced to 66, 75, 81 from 70, 82, 97 mV, respectively (Figure 5a). Interestingly, their Tafel slopes are further decreased to 37, 43, 56 mV dec^−1^ (Figure 5b). This change trend on the performance is consistent with some previous reports.^32^, ^44^, ^45^, ^46^ As we know, such a low overpotential (66 mV) to drive the 10 mA cm^−2^ and small Tafel slope means that Mo_2_C/G3‐NCS750 is one of Mo_2_C‐based electrocatalysts reported with best catalytic activity in alkaline electrolyte (Table S1, Supporting Information). From the Figure S16 (Supporting Information), the *R*_ct_ value of Mo_2_C/G3‐NCS750 is much smaller than that of Mo_2_C/NC750 and Mo_2_C/NCS750 under the same conditions, implying the faster electron transfer rate and higher catalytic activity of Mo_2_C/G3‐NCS750 electrocatalyst for HER, which agrees well with the analysis results of HER performance above‐mentioned. In addition, the polarization curves for Mo_2_C/G3‐NCS750 before and after 2000 CV cycles is almost overlap, indicating its long‐term durability in alkaline electrolyte (Figure S17, Supporting Information).

We demonstrate a simple, low cost, and scalable strategy for the fabrication of Mo_2_C‐based eletrocatalyst by a spray‐drying, and followed by annealing. As‐prepared Mo_2_C/G3‐NCS750 catalyst under the optimized synthetic condition exhibits excellent HER performance both in acidic and alkaline media, with a small overpotential, low Tafel slope, and long‐term durability, which is superior to most of the Mo_2_C‐based electrocatalysts reported previously (Table S1, Supporting Information) and close to the commercial Pt/C (20 wt%). The enhanced HER property is believed to attribute that the well‐defined porous microspherical structure, graphene wrapping, ultrasmall Mo_2_C nanocrystallinity, and nitrogen‐dopant offer many appealing features, such as a large exposed activity sites, improved electron transfer, fast charge transport, the enhanced interaction with H^+^ and low desorption energy of Mo—H bond. The facile preparation and excellent properties for G‐Mo_2_C/CS make this material very promising for practical application in hydrogen production. Futhermore, this method is expected to be generalized to prepare other metal carbides for electrocatalysis or energy storage and conversion devices, where exposed more active sites, and efficient ionic and electronic transport are critical.

## Experimental Section

*Preparation of the Catalyst*: Chitosan (1 g) was first dissolved deionized water (200 mL) containing acetate acid (2 mL). Second, AM (1 g) was dissolved into deionized water (10 mL), and then added into the 60 mL (5.0 mg mL^−1^) graphene oxide (GO) solution prepared according to the method reported previously by us.^47^ Subsequently, the above solution was sprayed into the chitosan solution and further stirred 12 h at room temperature to generate a mixture. Then, the powder was collected after spray‐drying the mixture, and further annealed under Ar at 750 °C for 3 h with a temperature ramping rate of 2 °C min^−1^ to yield the product (Mo_2_C/G3‐NCS750). Similarly, when the mass of GO is 0, 0.1, 0.5 g, Mo_2_C/NCS‐750, Mo_2_C/G1‐NCS750, and Mo_2_C/G5‐NCS750 can be readily obtained. For comparison, the Mo_2_C/G3‐NCS samples were carbonized at different carbonization temperatures such as 650, 850 °C, which were denoted as Mo_2_C/G3‐NCS650, Mo_2_C/G3‐NCS850, respectively. The Mo_2_C/NC750 is prepared using a similar procedure to Mo_2_C/NCS750, except that the solids were collected by centrifugation instead of spray‐drying. The mass ratio of AM and Chitosan is 2:1, 1:1, and 1:2, the corresponding products are denoted as Mo_2_C/0.5NC750, Mo_2_C/NC750, and Mo_2_C/2NC750, respectively.

*Electrochemical Measurements*: The electrochemical experiments for HER were carried out in a conventional three‐electrode cell using a CHI 660E at room temperature. Ag/AgCl (3 m KCl) and graphite rod were used as reference and counter electrodes, respectively in N_2_‐saturated 0.5 m H_2_SO_4_. Pt wire is used as reference electrode instead of graphite rod when the electrochemical experiments conducted in 1 m KOH solution. All potentials were corrected versus reversible hydrogen electrode (RHE) according to *E*_RHE_ = *E*_Ag/AgCl_ + *E*_Ag/AgCl_ + 0.059 × pH. 2 mg of the catalyst powder and 1 mg of black carbon were dispersed in a mixture of 400 µL of water, 100 µL of ethanol, and formed a catalyst ink after 1 h of sonication. Then, 10 µL of catalyst ink was pipetted onto the glassy carbon surface with a diameter of 3 mm (catalyst loading ≈0.57 mg cm^−2^). Then, 5 µL of 0.05 wt % Nafion solution was coated onto the electrodes and was dried at room temperature before measurement. The mass loading of referred Pt/C is same with the catalysts. The LSV was tested at a rate of 30 mV s^−1^ from 0.05 to −0.4 V versus RHE. The stability tests were carried out by repeating the potential scan from 0.05 to −0.4 V versus RHE for 2000 cycles. EIS measurements were carried out from 10^5^ to 0.01 Hz at −80 mV in acidic medium or −77 mV in alkaline medium versus RHE. The double‐layer capacitances (*C*_dl_) were obtained through cyclic voltammograms (CV) curves which were performed at scan rates varying from 20 to 180 mV s^−1^ in the potential region from 0.2 to 0.4 V versus RHE.

## Conflict of Interest

The authors declare no conflict of interest.

## Supporting information

Supplementary
