# Supplementary material for: Molybdenum Carbide Nanoparticles Coated into the Graphene Wrapping N‐Doped Porous Carbon Microspheres for Highly Efficient Electrocatalytic Hydrogen Evolution Both in Acidic and Alkaline Media
Source: Adv Sci (Weinh). 2018 Jan 3;5(3):1700733. doi: 10.1002/advs.201700733 (PMC5867049; doi:10.1002/advs.201700733)
Supplement: Supplementary file 1 — Supplementary [file ADVS-5-1700733-s001.pdf]

Copyright WILEY-VCH Verlag GmbH & Co. KGaA, 69469 Weinheim, Germany, 2018.

ADVANCED SCIENCE
Open Access

Supporting Information

for *Adv. Sci.,* DOI: 10.1002/advs.201700733

Molybdenum Carbide Nanoparticles Coated into the Graphene Wrapping N-Doped Porous Carbon Microspheres for Highly Efficient Electrocatalytic Hydrogen Evolution Both in Acidic and Alkaline Media

Huifang Wei, Qiaoya Xi, Xi'an Chen, Daying Guo, Feng Ding, Zhi Yang, Shun Wang, Juan Li, and Shaoming Huang**

Molybdenum carbide nanoparticles coated into the graphene wrapping N-doped porous carbon microspheres for highly efficient electrocatalytic hydrogen evolution both in acidic and alkaline media

Huifang Wei, Qiaoya Xi, Xi'an Chen*, Daying Guo, Feng Ding, Zhi Yang, Shun Wang, Juan Li, Shaoming Huang*

H. F. Wei, Q. Y. Xi, Dr. X. Chen, D. Y. Guo, F. Ding, Prof. Z. Yang, Prof. S. Wang, J. Li, Prof. S. M. Huang

Key Laboratory of Carbon Materials of Zhejiang Province, College of Chemistry and Materials Engineering, Wenzhou University, Wenzhou 325035, P.R. China.

Prof. S. M. Huang

School of Materials and Energy, Guangdong University of Technology, Guangzhou, Guangdong 510006 P.R. China.

E-mail: xianchen@wzu.edu.cn (Xi'an Chen), smhuang@wzu.edu.cn (Shaoming Huang)

Materials and regents

All the reagents were analytical grade and used without further purification. Ammonium molybdate tetrahydrate (AM), chitosan(CS), ethanol and acetate acid were purchased form Aladdin. Nafion perfluorinated ion-exchange resin solution (5 wt%), Pt/C (20 wt %) were offered by Sigma-Aldrich.

Materials characterization

SEM images were obtained with a Nova NanoSEM 200 scanning electron microscope (FEI, Inc.). TEM, HRTEM images were recorded with a JEOL2100 instrument. Powder XRD was performed on a Bruker D8 Advance X-ray diffractometer using CuKa radiation (λ= 0.15418 nm) at a scanning rate of 4°min^{-1} in the 2θ range from 10° to 80°. X-ray photoelectron spectroscopy (XPS) measurements were conducted with an ultrahigh vacuum setup, equipped with a monochromatic Al Ka X-ray source and a high resolution Thermo ESCALAB 250 analyzer. The content of Mo_2C on the composite was tested using a TG/DTA thermogravimetric analyzer (Diamond PE) under an O_2 atmosphere at a heating rate of 10 °C min^{-1} from room temperature to 800 °C, with a flow rate of 100 mL min^{-1}. The nitrogen adsorption/desorption isothermal curves were obtained on a Micromeritics ASAP 2020 instrument.

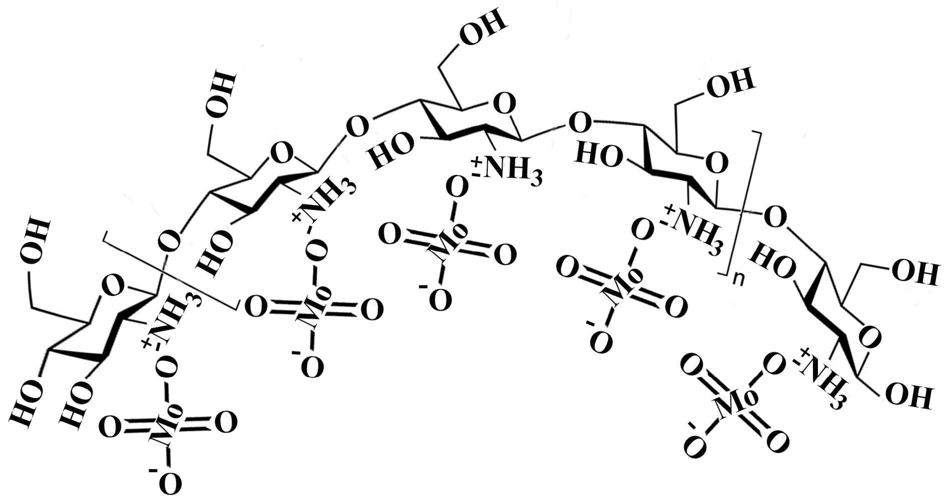

Fig. S1 Schematic illustration of electrostatic interaction between AM and chitosan.

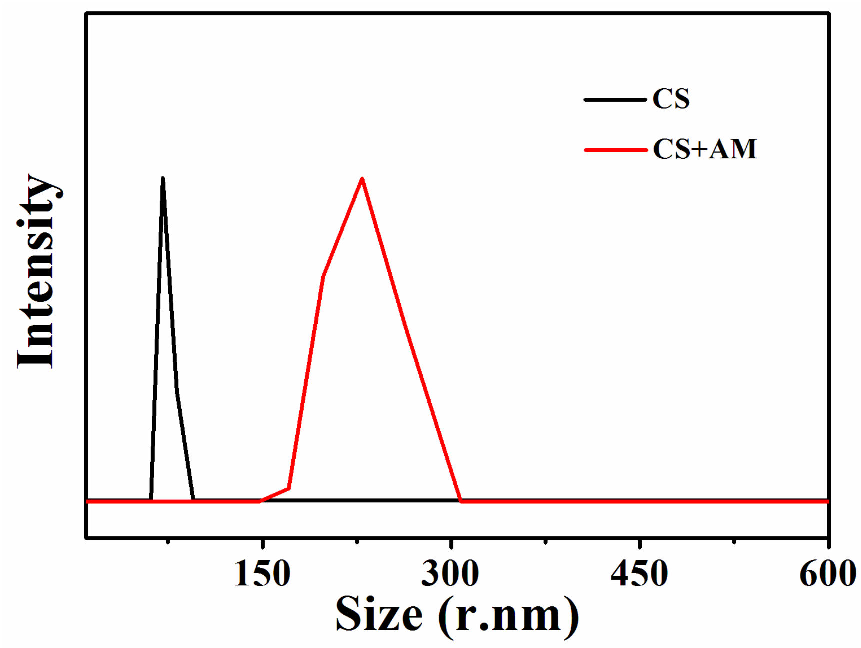

Fig. S2 The particle size analysis of chitosan (CS) solution and the mixture of CS and AM in a laser particle size analyzer Zetasizer ZS90.

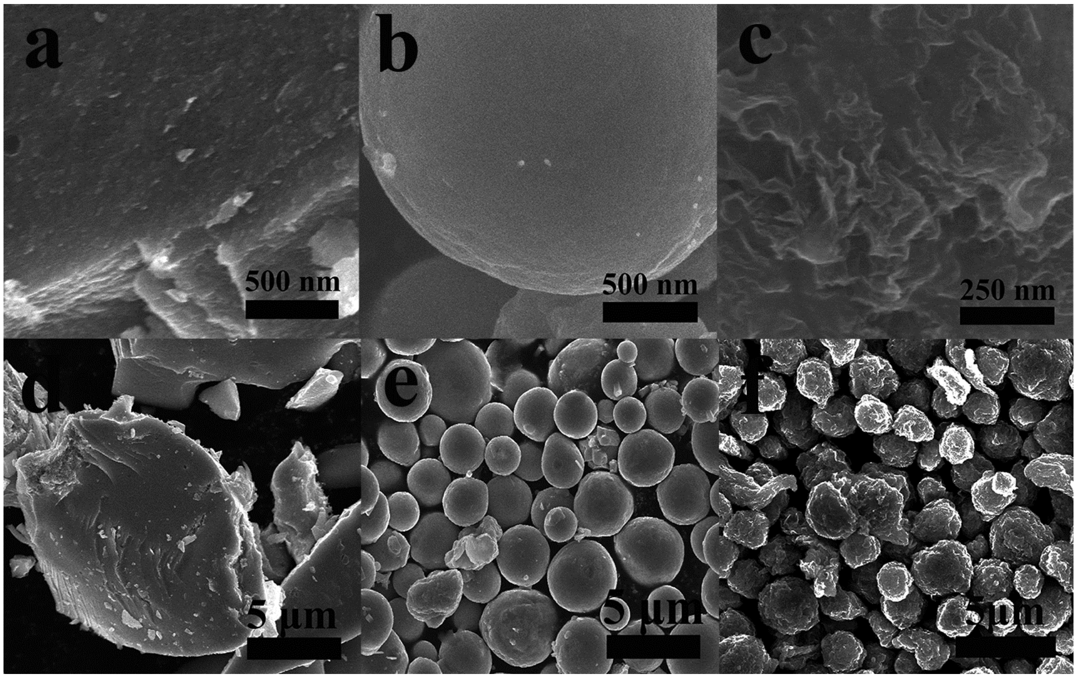

Fig. S3 SEM imagesat different magnifications of (a, d) Mo_2C/NC750, (b, e) Mo_2C/NCS750, (c, f) Mo_2C/G3-NCS750.

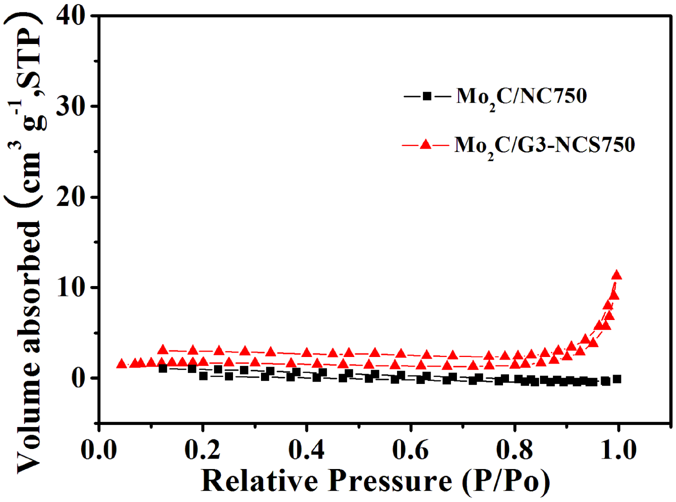

Fig. S4 The nitrogen adsorption/desorption isothermal curves of Mo_2C/G3-NCS750 and Mo_2C/NC750

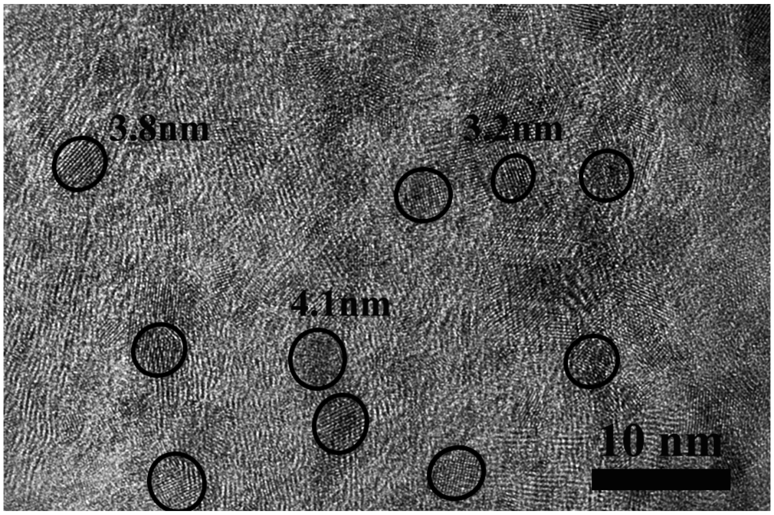

Fig. S5 the HRTEM image of Mo_2C/G3-NCS750

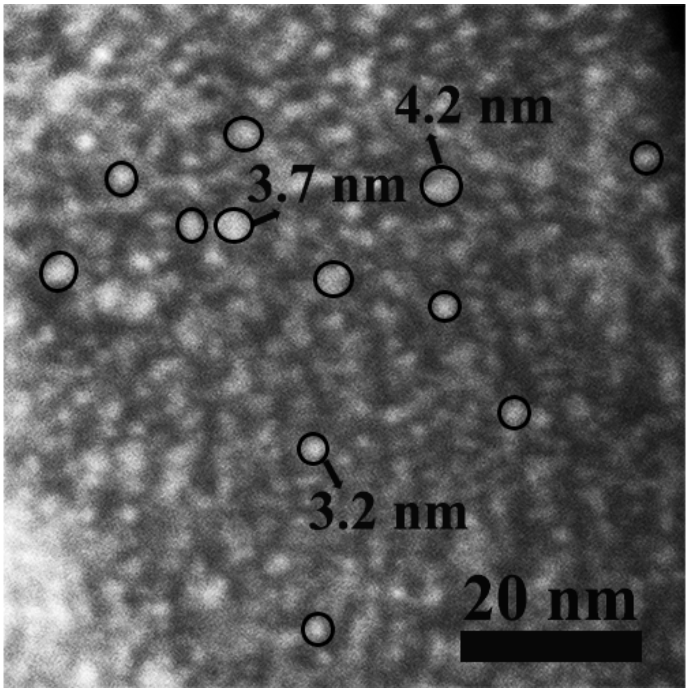

Fig. S6 the bright and dark field TEM image of Mo_2C/G3-NCS750

Particle size calculation:

Scherrer 公式： $D = \frac{k\lambda}{\beta \cos\theta}$

λ=0.15418 nm

$\beta = FWHM \times \frac{\pi}{180}$

$\theta = Theta \times \frac{\pi}{180}$

k=0.89

Mo_2C/G3-NCS750

(101)

$$D = \frac{k\lambda}{\beta \cos\theta} = \frac{0.89 \times 0.15418}{1.843 \times \frac{\pi}{180} \times \cos(19.8 \times \frac{\pi}{180})} = 4.5nm$$

（110）

$$D = \frac{k\lambda}{\beta \cos\theta} = \frac{0.89 \times 0.15418}{1.957 \times \frac{\pi}{180} \times \cos(30.95 \times \frac{\pi}{180})} = 4.7nm$$

Average size: $\frac{4.5 + 4.7}{2} = 4.6(\text{nm})$

Mo_2C/G3-NCS850

(101)

$$D = \frac{k\lambda}{\beta \cos\theta} = \frac{0.89 \times 0.15418}{1.164 \times \frac{\pi}{180} \times \cos(19.8 \times \frac{\pi}{180})} = 7.2nm$$

(110)

$$D = \frac{k\lambda}{\beta \cos\theta} = \frac{0.89 \times 0.15418}{1.475 \times \frac{\pi}{180} \times \cos(30.95 \times \frac{\pi}{180})} = 6.2nm$$

Average size: $\frac{7.2 + 6.2}{2} = 6.7(\text{nm})$

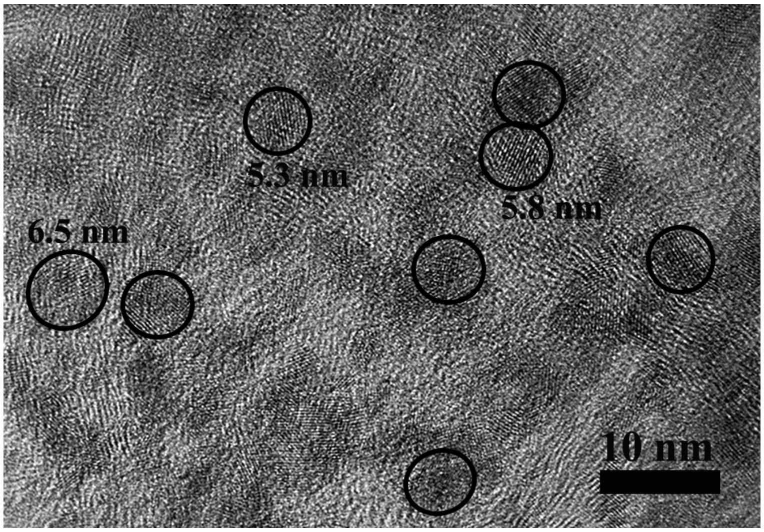

Fig. S7 the HRTEM image of Mo_2C/G3-NCS850

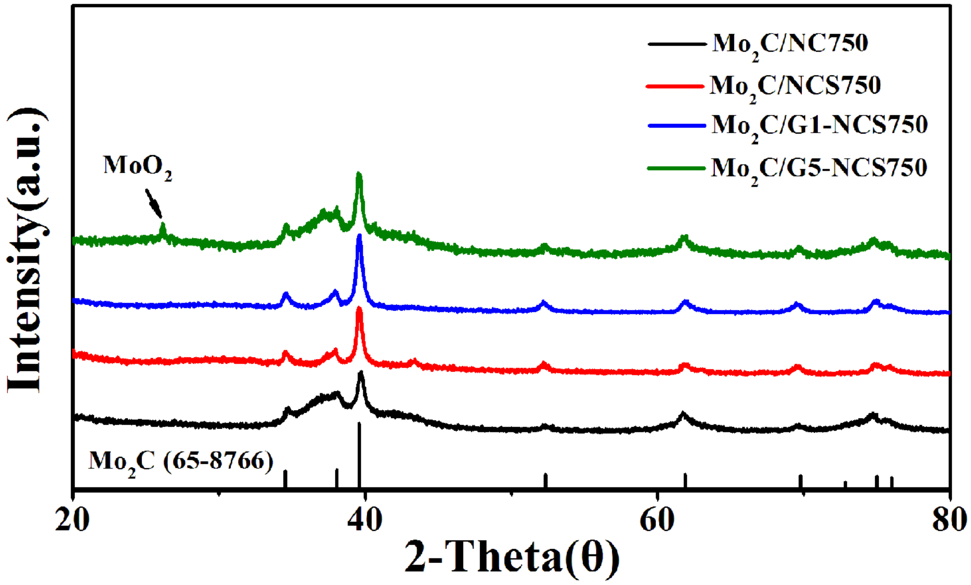

Fig. S8 XRD patterns of Mo_2C/NC750, Mo_2C/NCS750, Mo_2C/G1-NCS750 and Mo_2C/G5-NCS750.

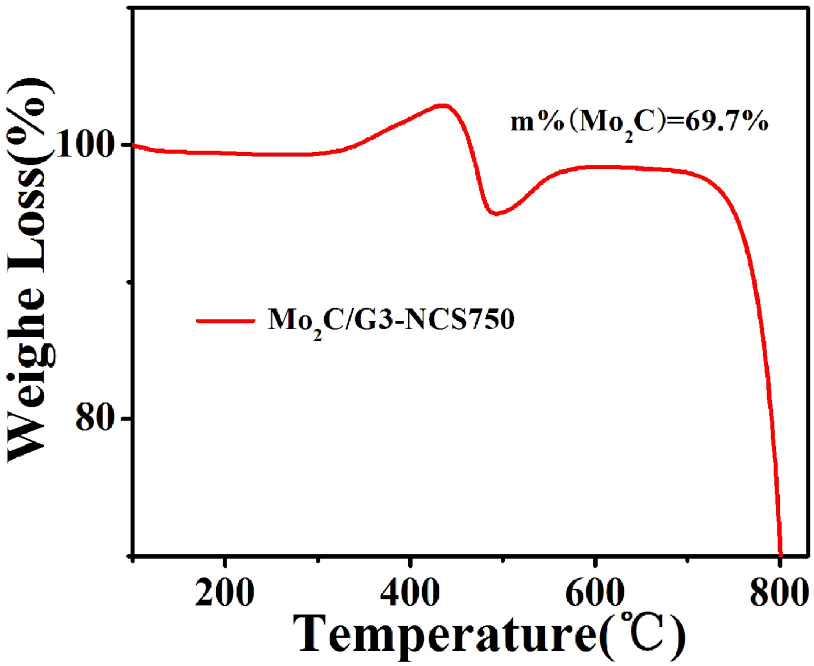

Fig. S9 TGA curves for Mo_2C/G3-NCS750.

As shown in TGA curve, all Mo_2C nanoparticles were oxidized to MoO_3 during the TGA measurement in oxygen atmosphere, followed by the combustion of carbon, and converts to only MoO_3 after 600 ℃. The weight percent of Mo_2C in Mo_2C/G3-NCS750 is estimated according to the following equation:

$$m\% (Mo_2C) = \text{residual mass} * M(Mo_2C)/2M(MoO_3)$$

$$= 98.4 \text{ wt.\%}*204/(2*144) = 69.7 \text{ wt.\%}$$

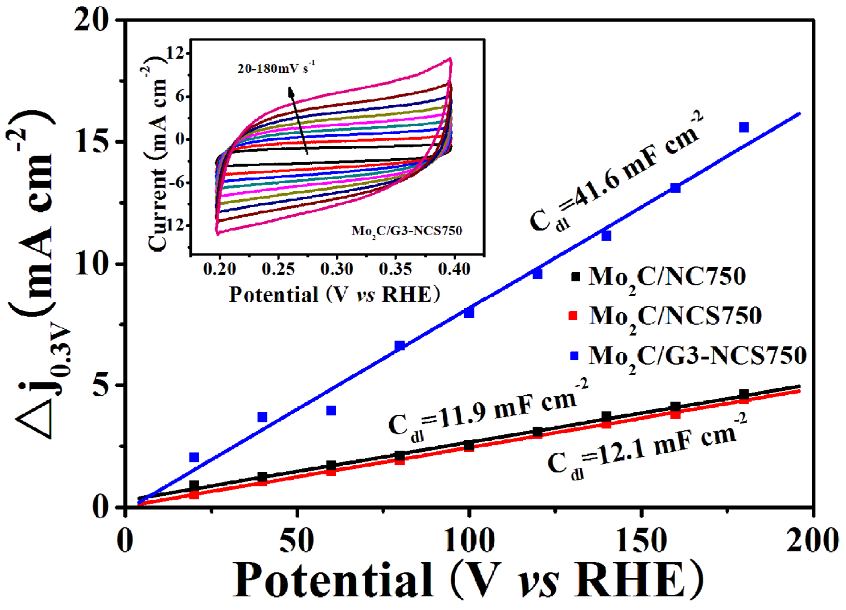

Fig. S10 Capacitive current at 0.3 V as a function of scan rate for Mo_2C/NC750, Mo_2C/NCS750 and Mo_2C/G3-NCS750 ($\triangle j_0=j_a-j_c$; inset: CVs for Mo_2C/G3-NCS750 with different rates from 20 to 180 mV s^{-1} in the potential range of 0.2-0.4 V).

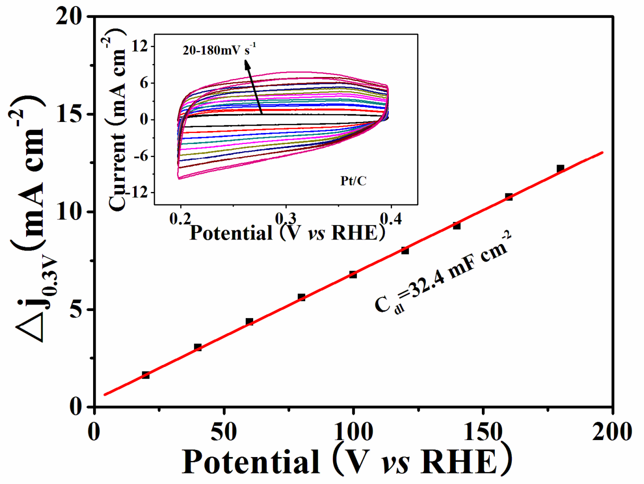

Fig. 11 Capacitive current at 0.3 V as a function of scan rate for Pt/C ($\triangle j_0=j_a-j_c$; inset: CVs for Pt/C with different rates from 20 to 180 mV s^{-1} in the potential range of 0.2-0.4 V).

ECSA calculation:

$$C = \frac{k}{2} \times \frac{1}{m} = \frac{41.6\,mF/cm^2}{2} \times \frac{1}{0.57mg/cm^2} = 36.5F/g$$

where C is the specific capacitance of Mo_2C/G3-NCS750, k is the fitting slope, m is the catalyst areal loading.

Then, we can calculate its ECSA by assuming a standard value of 40 μF/cm^2:

$$ECSA = \frac{C}{40\mu F/cm^2} = \frac{36.5F/g}{40\mu F/cm^2} = 91.3cm^2/g$$

Pt/C

$$C = \frac{k}{2} \times \frac{1}{m} = \frac{32.4\,mF/cm^2}{2} \times \frac{1}{0.57mg/cm^2} = 28.4F/g$$

$$ECSA = \frac{C}{40\mu F/cm^2} = \frac{28.4F/g}{40\mu F/cm^2} = 71cm^2/g$$

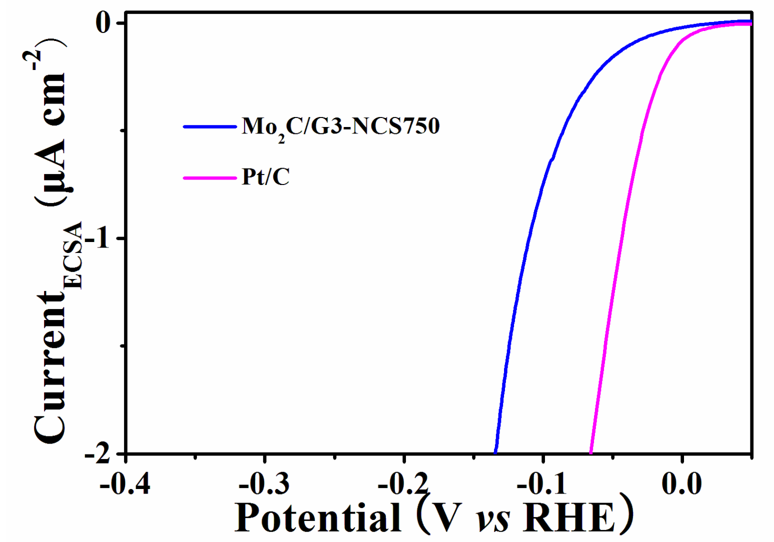

Fig. 12 LSV curves normalized by ECSA for Mo_2C/G3-NCS750 and Pt/C.

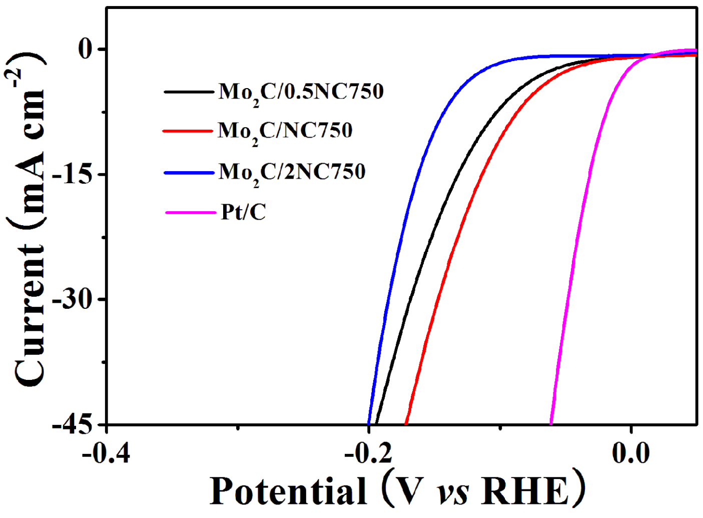

Fig. S13 LSV curves of Mo_2C/0.5NC750 (2:1), Mo_2C/NC750(1:1), Mo_2C/NC750 (1:2) and Pt/C

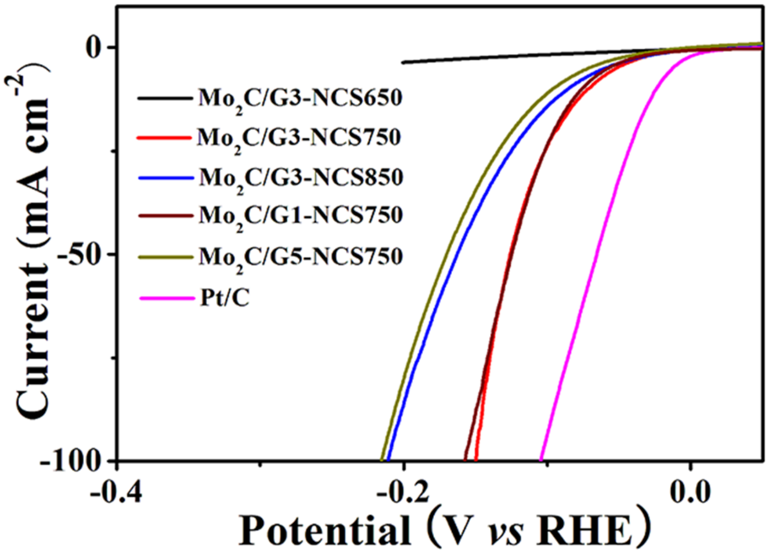

Fig. S14 LSV curves of Mo_2C/G3-NCS650, Mo_2C/G3-NCS750, Mo_2C/G3-NCS850, Mo_2C/G1-NCS750, Mo_2C/G5-NCS750 with a scan rate of 30 mV s^{-1} in 0.5 M H_2SO_4 solution.

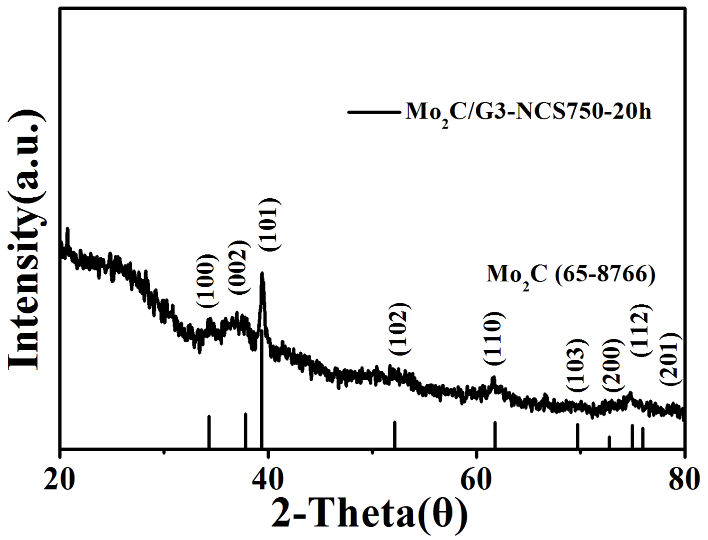

Fig. S15 XRD patterns of Mo_2C/G3-NCS750 after chronoamperometric test for 20 h in acidic electrolyte.

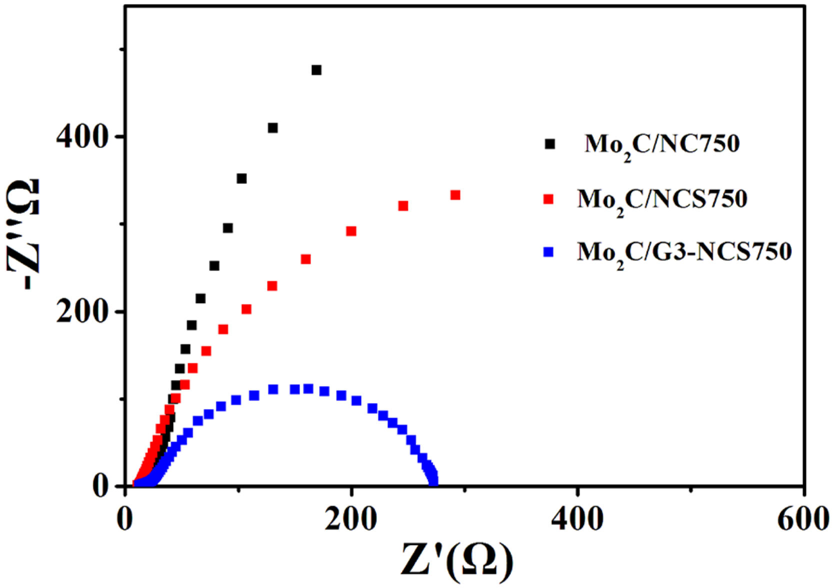

Fig. S16 EIS Nyquist plots collected at a bias voltage of -77 mV for Mo_2C/NC750, Mo_2C/NCS750 and Mo_2C/G3-NCS750 in 1 M KOH solution.

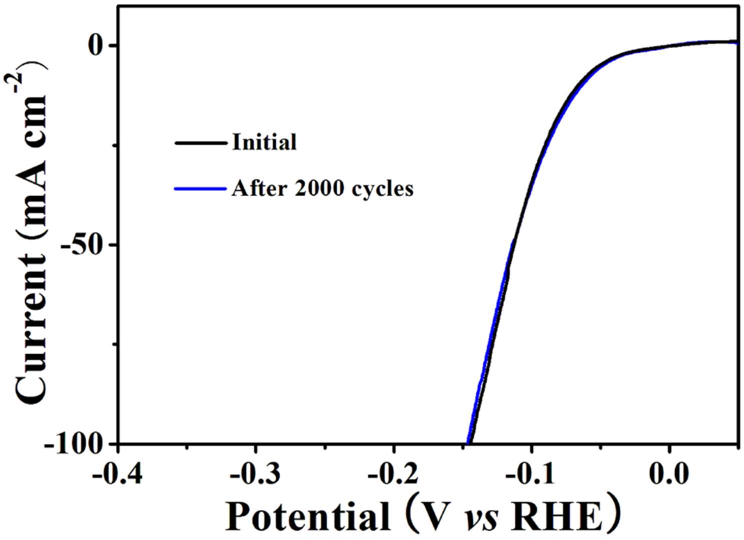

Fig. S17 LSV curves initial and after 2000 CV of Mo_2C/G3-NCS750 in 1 M KOH solution.

Table S1 Comparison of HER performance for Mo_2C/G3-NCS750 with other Mo-based electrocatalysts.

Catalyst	Electrolyte	Loading density /mg cm^{-2}	η10/mV	Tafel/mV dec^{-1}	Reference
0.3G-Mo_2C/NCS	0.5 M H_2SO_4<br>1 M KOH	0.57	70<br>66	39<br>37	**This work**
Mo_2C	1 M H_2SO_4<br>1 M KOH	1.4<br>0.8	210<br>190	56<br>54	Angew. Chem. Int. Ed. 2012, 51, 12703.
Mo_2C/CNT	0.1 M $HClO_4$	2	152	55.2	Energy Environ. Sci. 2013, 6, 943
Mo_2C-RGO	0.5 M H_2SO_4	0.285	130	57.3	Chem. Commun. 2014, 50, 13135
Mo_2C@NC	0.5 M H_2SO_4	0.25	78	41	Angew. Chem. 2015, 54, 10752
Mo_2C NPs	0.5 M H_2SO_4<br>1 M KOH	0.102<br>0.102	198<br>176	56<br>58	J. Mater. Chem. A 2015, 3, 8361
MoC_x octahedra	0.5 M H_2SO_4<br>1 M KOH	0.8<br>0.8	142<br>151	53<br>59	Nature Commun. 2015, 6, 6512
MoCN	0.5MH_2SO_4	0.4	145	46	J. Am. Chem. Soc. 2015, 137, 110

N,P/ Mo_2C@C	0.5MH_2SO_4	0.9	141	56	ACS Nano 2016, 10, 8851
Mo_2C@NPC/NPRGO	0.5 M H_2SO_4	0.14	34	33.6	Nature Commun. 2016, 7, 11204
Mo_2C-GNR	0.5 M H_2SO_4	0.28	167	63	ACS Sustainable Chem. Eng. 2016, 4, 6313
3DHP-Mo_2C	0.5 M H_2SO_4	0.28	97	60	Small 2016, 12, 2859
Mo_2C/NPCNFs	0.5 M H_2SO_4	0.4	134	68	Carbon 2016.12.081
Mo_2C/NCF	0.5 M H_2SO_4<br>1 M KOH	0.28	144<br>100	55<br>65	ACS Nano 2016, 10 , 11337
MCC-3	0.5 M H_2SO_4	1	175	66	Small 2017, 1701246
MoP/Mo_2C@C	0.5 M H_2SO_4	0.453	89	45	ACS Appl. Mater. Interfaces 2017, 9, 16270
Mo_2C/C	0.5 M H_2SO_4	0.84	135	75.1	J. Mater. Chem. A 2017, 5, 4879
Mo_2C@2D-NPC	0.5 M H_2SO_4	0.247	86	62	ACS Nano 2017, 11 , 3933
MoP/Mo_2C@C	0.5 M H_2SO_4	0.453	89	45	ACS Appl. Mater. Interfaces 2017, 9, 16270
Mo_2C@NPC-4	0.5 M H_2SO_4	0.265	144	52.5	J. Mater. Chem. A 2017, 5, 5178
P-Mo_2C@C nanowires	0.5 M H_2SO_4	1.30	89	42	Energy Environ. Sci. 2017, 10, 1262
Mo_2C nanoribbon/N-G film	0.5 M H_2SO_4	(0.5*0.5cm)	162	57	J. Mater. Chem. A 2017, 5, 12027
MoC@NC nanoribbon	0.5 M H_2SO_4<br>0.1 M KOH	0.385	24 (onset)<br>36 (onset)	54<br>51	ACS Appl. Mater. Interfaces 2017, 9, 24608
Mo_2C–GNR	0.5 M H_2SO_4<br>0.1 M KOH	0.57	152<br>121	69<br>59	ACS Nano 2017, 11, 384
